# Progress feedback in children and adolescents with internalizing and externalizing symptoms in routine care (OPTIE study): study protocol of a randomized parallel-group trial

**DOI:** 10.1186/s12888-021-03502-w

**Published:** 2021-10-15

**Authors:** Christopher Hautmann, Jana Rausch, Nina Geldermann, Felix Oswald, Danny Gehlen, Martin Hellmich, Kerstin Daniela Rosenberger, Christina Samel, Katrin Woitecki, Daniel Walter, Julia Adam, Lydia Dachs, Hildegard Goletz, Joya Halder, Claudia Kinnen, Kristina Mücke, Janina Otte, Daniela Perri, Christiane Rademacher, Stephanie Schürmann, Paula Viefhaus, Tanja Wolff Metternich-Kaizman, Manfred Döpfner

**Affiliations:** 1 School for Child and Adolescent Psychotherapy (AKiP), Faculty of Medicine and University Hospital Cologne, University of Cologne, Cologne, Germany; 2 Department of Child and Adolescent Psychiatry, Psychosomatics and Psychotherapy, Faculty of Medicine and University Hospital Cologne, University of Cologne, Cologne, Germany; 3 Institute of Medical Statistics and Computational Biology, Faculty of Medicine and University Hospital Cologne, University of Cologne, Cologne, Germany

**Keywords:** Progress feedback, Routine outcome monitoring, Feedback-informed treatment, Behavior therapy, Children and adolescents, Internalizing, Externalizing, Randomized controlled trial

## Abstract

**Background:**

Progress feedback provides therapists with progress notes on a regular basis through the continuous assessment of participants throughout their treatment (e.g., symptoms, therapeutic alliance). While for adults the evidence base has increased over the years, progress feedback in the therapy of children and adolescents has not been sufficiently investigated. This manuscript describes the trial protocol of the OPTIE study: a randomized trial that tests the efficacy of a progress feedback system in children and adolescents under conditions of routine care.

**Methods:**

The study is based on a randomized parallel-group trial with two treatment groups (routine, feedback) at an outpatient unit of a university hospital. The target sample size is 439 families consisting of children and adolescents aged 6 to17 years old with internalizing and/or externalizing symptoms. Both the patients and the therapists are independently assigned to the treatment groups by stratified block randomization. In both treatment groups patients receive routine care behavioral therapy for a study-related 12 months; additionally, in the feedback group, a progress feedback system with three components is applied (monitoring, report, and supervision). For three informants (caregiver, child [≥ 11 years], therapist) surveys are conducted every 6 weeks (e.g., symptoms, goals, motivation). For both treatment groups, comparison data is collected at baseline and at six and 12 months after the beginning of the intervention (pre, inter, post), and includes five informants (blinded clinician, therapist, caregiver, child [≥ 11 years], teacher).

**Discussion:**

The OPTIE study will contribute to the evidence base of progress feedback in children and adolescents and has the potential to uncover treatments’ effects in the small to medium range. Noteworthy features are the inclusion of children younger than 10 years old and the consideration of a blinded clinician rating.

**Trial registration:**

German Clinical Trials Register (DRKS) DRKS00016737 (https://www.drks.de/DRKS00016737). Registered 17 September, 2019.

Psychiatric disorders in children and adolescents are prevalent in society [1]. Psychotherapy has shown to be effective across a wide range of different disorders [2]; however, there are still limitations. Some patients do not sufficiently improve during therapy or their condition worsens. In adults, deterioration rates of about 8% have been reported [3] and in children and adolescents rates of about 24% have been found [4]. Additionally, about a quarter of all patients drop out during cognitive behavioral therapy [5, 6].

Progress feedback aims to improve therapy outcomes by supporting the therapist with additional information about the therapy through the continued assessment of the patient’s progress throughout treatment. This concept has often been attributed to the work of Ken Howard [7]: under the term patient-focused research a paradigm “that is concerned with the monitoring of an individual’s progress over the course of treatment and the feedback of this information to the practitioner, supervisor, or case manager” was introduced (p.1059) [7]. In literature, different terms have been used in relation to this concept, including routine outcome monitoring [8], measurement feedback system [9], patient-reported outcome measures [10], measurement-based care [11], feedback-informed treatment [12], and progress monitoring [13].

Progress feedback has the potential to help improve information processing performed by psychotherapists. It may be that in therapy deteriorations are difficult to discern and that predictions about a patient’s progress are hard to make, especially in cases involving an undesirable treatment outcome [14]. It has also been shown that work experience has a rather small effect on decision-accuracy [15]. The weak association between work experience and therapeutic expertise may in part be explained by an information deficit among therapists as well as their difficulties in estimating the effects of an intervention on the patient [16]. Progress feedback aims to mitigate this deficit and help therapists to adapt treatment planning.

Several suggestions have been proposed to enable progress feedback systems to be useful and effective [17, 18]. They should be brief and used regularly, they should be orientated toward the needs and requirements of the patient and the clinician, and, whenever possible, they should consider multiple perspectives (e.g., child, parent, and therapist). The instruments used for monitoring should have sound psychometric properties (e.g., sensitivity) and, alongside symptoms, they should include further relevant process parameters (e.g., therapy motivation, therapeutic relationship). The feedback should be given in a timely manner, soon after the assessment, in writing. In addition to the diagnostic findings, recommendations about future therapy should also be included.

The efficacy of progress feedback in adults has been extensively investigated and findings have been reported in several meta-analyses [19–25] and review articles [8, 10, 26–29]. Progress feedback has been positively evaluated in that it improves communication between the therapist and the patient and helps adapt the treatment to the needs of the patient [8, 29]. Regarding the improvement of mental health, small effect sizes (Cohen’s *d* or Hedge’s *g*) have been reported, for example, 0.07 (not significant) [19], 0.10 [20], 0.14 [22], 0.15 [23], and 0.27 [24]. However, moderating factors are likely to be present and some patients may profit from progress feedback more than others. Patients at risk of a negative outcome – often labelled not-on-track – have received particular attention. Reported effect sizes (Cohen’s *d* or Hedge’s *g*) for not-on-track patients are 0.17 [23], 0.22 [19], 0.28 (or higher depending on the subsample) [21], and 0.33 [22].

For psychotherapy in children and adolescents, however, the evidence base for progress feedback is much less developed. In 2012, Carlier et al. could not identify a single randomized trial for this age group [8]. In the meantime, meta-analyses have become available [30, 31]. Tam and Ronan identified 12 studies involving youths aged 10 to 19 years old with mental health problems (randomized controlled trials, quasi-experiments, single-subject experiments) [31]. Data from four randomized controlled trials was available and revealed a small effect size (Hedge’s *g*) of 0.20 in favor of the progress feedback. For a Cochrane review, Bergman et al. considered six randomized trials involving children and adolescents [30]. The results for the youth reports were too heterogenous to be pooled, and for the therapist, parent, and teacher reports either no or only slight differences were ascertained. In addition, only one study included a blinded rating and none of the studies considered children younger than 10 years old. The authors concluded that no firm conclusions could be drawn about the efficacy of progress feedback in children and adolescents at that time and more high-quality studies were needed [30].

In the following, the study protocol of the OPTIE study (Optimierung des Therapieerfolgs durch Prozessfeedback in der Verhaltenstherapie von Kindern und Jugendlichen mit internalen und externalen Störungen [Optimizing treatment outcomes through progress feedback in cognitive behavioral therapy for children with internalizing and externalizing disorders]) is described (version 4 from 14 December, 2020). The main aim of the study is to test the efficacy of a progress feedback system for children and adolescents aged 6 to 17 years old with internalizing and externalizing symptoms in a randomized parallel-group trial. The study is conducted under conditions of routine care and includes ratings from five different informants (blinded clinician, therapist, caregiver, child [≥ 11 years], teacher). A large clinical sample of 439 families is intended. The main hypothesis is that behavior therapy in combination with progress feedback is more effective in reducing patients’ symptoms than behavior therapy alone.

## Methods

### Study site and organization

The study takes place at AKiP (Ausbildungsinstitut für Kinder- und Jugendlichenpsychotherapie an der Uniklinik Köln [School for Child and Adolescent Psychotherapy at the University Hospital Cologne]). AKiP is engaged in the teaching and applied training of post-graduate students in behavioral therapy for children and adolescents. Part of AKiP is an outpatient unit with about 900 new admissions per year of children and adolescents. The study therapies are undertaken at the AKiP outpatient unit.

**Fig. 1:**
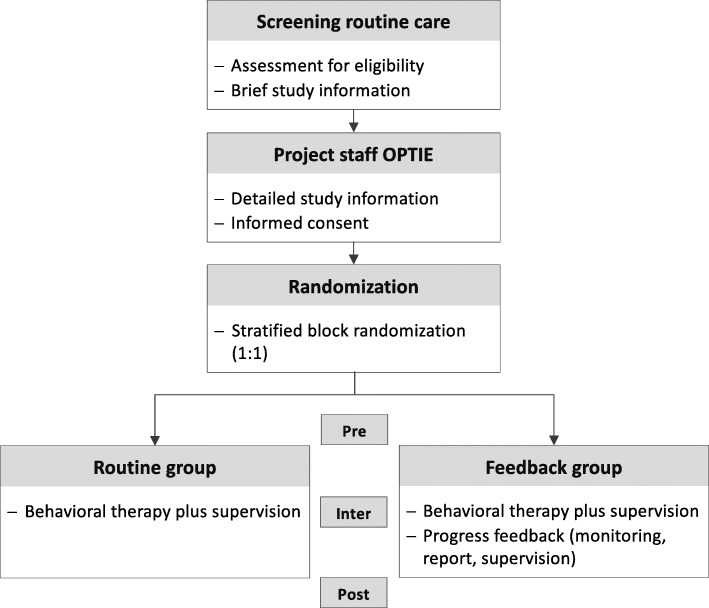
Participant flow from the screening procedure to the last assessment. Note. Pre, inter, and post refer to the assessments at baseline and at 6 and 12 months after the beginning of the therapy which include a blinded clinical interview with the caregiver as well as questionnaires for several respondents (blinded clinician, therapist, caregiver, child [≥ 11 years], teacher)

All patients who apply for therapy will be tested by the AKiP staff during the initial clinical interview for their eligibility for the study (see Fig. 1). Patients who fulfil the criteria and who are interested in the study will be referred to the project staff. The project staff has the main responsibility for all study-specific processes (e.g., recruitment, assessment, monitoring). This includes confirming that all ethical requirements are fulfilled and maintaining response rates for the assessments throughout the study (e.g., reminders). After the randomization procedure, patients are allocated to a treatment group.

### Study therapists

The study therapists are post-graduate students trained at AKiP. In total there are around 150 therapists in training who provide psychotherapy under supervision as part of their practical education at the AKiP outpatient unit. All therapists in training hold a master’s degree or the German Diplom in various professions (e.g., psychology, pedagogy, social work, or rehabilitation science).

### Inclusion criteria

The scope of the inclusion criteria is limited to a minimum, since the therapies are undertaken under the conditions of routine care: (1) children and adolescents aged 6 to 17 years old, (2) internalizing and/or externalizing problems, (3) indication for outpatient psychotherapy, and (4) sufficient German language skills of at least one guardian.

The criteria are all assessed by a clinician at the initial screening of the patient. The second inclusion criterion is based on a clinical rating operationalized by items of the German CASCAP-D (Psychopathologisches Befund-System für Kinder und Jugendliche [Clinical assessment-scale for child and adolescent psychopathology]) [32]. For the Externalizing dimension, items of the following CASCAP domains are used (number of items considered for the study): Disturbances of Conduct (8 items) and Disturbances of Drive, Attention, and Impulse Control (3 items). For the Internalizing dimension the domains of the items are as follows: Anxiety Disorders (7 items), Obsessive-Compulsive Disturbances (2 items), and Disturbances of Mood and Affect (10 items). All items are rated on a four-point Likert-type scale (0 = *age appropriate/normal*, 1 = *mild*, 2 = *marked*, 3 = *severe*). This inclusion criterion is considered fulfilled if at least one item of the Externalizing or Internalizing dimension is rated 2 or 3 during the clinical expert rating.

### Study design

The progress feedback system is evaluated by a randomized parallel-group design with two treatment arms (routine, feedback).

### Randomization

Both patients and therapists are independently assigned to the two treatment conditions by stratified block randomization (block size: permuted block randomization of varying length; randomization ratio: 1:1). For patients, the stratification factors of gender (male, female) and the symptom domain (internalizing, externalizing, both) are considered. For therapists, the stratification is based on the factors of gender (male, female) and age (≤ 30, > 30). To maintain internal validity, therapists are randomized only once upon entry to the study and working with a second patient does not change their initial treatment group. Random assignment is done using the 24/7 Internet service ALEA (FormsVision BV, Abcoude, NL) which is maintained by the Institute of Medical Statistics and Computational Biology at the Faculty of Medicine and University Hospital Cologne, University of Cologne.

Two forms of randomization for patients are permitted: Regular randomization means that patients assigned to the routine group receive a therapist from the routine group and patients assigned to the feedback group receive a therapist from the feedback group. However, under conditions of routine care, the volume of patients is high and the initial study experience has shown that sometimes study therapists are unavailable. Thus, to ensure that patients can be enrolled and treated without delay, forced randomization is also permitted in exceptional cases [33]. This means that the patient is assigned post-hoc to an available study therapist from the other treatment condition and is also analyzed in this group (intention-to-treat).

### Treatment arms

The study has the two treatment arms of routine group and feedback group. The study-related duration of the intervention in both arms is 12 months. However, as the study is conducted under conditions of routine care, it is at the discretion of the therapist to prolong or shorten the treatment duration depending on the needs of the patient and the treatment planning. In cases where the treatment is terminated early or prolonged all study-specific interventions are stopped (i.e., the progress feedback system), but the assessments are collected as scheduled.

#### Routine group

Families assigned to the routine group receive guideline-based behavioral therapy. Because the therapies are conducted by therapists in training, regulatory standards require that (on average) every fourth session is supervised by a licensed supervisor. Regarding the form and the content of the therapies there are no study-related regulations.

#### Feedback group

As in the routine group, families of the feedback group also receive guideline-based behavioral therapy. In addition, the study progress feedback system is offered.

### OPTIE progress feedback system

The progress feedback system is applied to the feedback group only and consists of three major components: monitoring, report, and supervision. During the 12-month intervention period, the participants are invited to answer brief online surveys every 6 weeks (e.g., symptoms, goal attainment). The results are reported to the therapist and the supervisors in the form of a progress report. Therapists are requested to take part in five additional sessions of supervision dedicated to the progress feedback system to discuss the progress reports and to adapt the treatment planning when necessary.

#### Participants and participation behavior

For the progress feedback system three participants are considered: (1) the caregiver, (2) the child, and (3) the therapist. The term caregiver is used here for a biological parent or guardian who accompanies the child during therapy. If two caregivers actively participate in the treatment, only one of them must be assigned to the progress feedback system. It may be that only the child is involved in the therapeutic process. If the child does not want their caregivers to be part of the therapy the caregivers will not participate in the progress feedback system. The patient’s rating is only collected from children 11 years and older. Ratings from younger children are not addressed for psychometric reasons (e.g., reliability, validity) and because the questionnaires of the progress feedback system are conceived for older children. The third rating comes from the therapist and the report is collected with every therapy.

At the start of the trial, a monitoring system was established to control the participation behavior of the respondents and to increase adherence. In cases of nonresponse to the online surveys, the project staff contacts the participants to offer support (e.g., reminder text message). Additionally, with each progress report therapists and supervisors are notified about the participation behavior.

#### Data collection and report creation

In general, the data of the progress feedback system is collected via the software REDCap (Research Electronic Data Capture) [34]. REDCap is a secure web application for the management of clinical trial data, including tools for building and managing online surveys. For completion of the surveys in the progress feedback system all participants receive an e-mail containing a link to the online surveys. If participants do not have the technical requirements for the online survey, paper-pencil questionnaires can also be used and sent back to the project team for data entry.

The data from the REDCap database are exported and subsequently processed by R [35]. The progress reports are generated as PDF files and are produced by Sweave [36], a framework that allows the combination of R and LaTeX. Therapists and supervisors then receive the progress reports by e-mail.

#### Frequency

The first survey is conducted before the start of therapy and surveys two to nine are scheduled for every 6 weeks after the first therapy session, resulting in nine surveys in total over the 12-month intervention period (see Table 1). Caregivers and children (≥ 11 years) are scheduled to complete all surveys. The assessments by the therapists are scheduled to start with the second survey. All participants have 10 days to complete each survey.

**Table 1:** Measures of the OPTIE progress feedback system for caregiver, child (≥ 11 years) and therapist ratings

Measure	Domain	Survey		
		1	2	3–9
Caregiver				
BPM/6–18	Symptoms – child		X	X
ZIEBO	Goal attainment			X
MYTS	Treatment motivation	X	X	X
TAQR	Therapeutic alliance		X	X
Child (≥ 11 years)				
BPM/6–18	Symptoms – child		X	X
ZIEBO	Goal attainment			X
MYTS	Treatment motivation	X	X	X
TAQS	Therapeutic alliance		X	X
YCIS v.2	Treatment impact		X	X
Therapist				
TAQR	Therapeutic alliance		X	X
OAS	Adherence		X	X

*Note*. The first survey is conducted before the beginning of the therapy, and surveys two to nine begin 6 weeks after the first therapy sessions and reoccur every 6 weeks. *BPM/6–18* Brief Problem Monitor for Ages 6–18 [37, 38], *ZIEBO* German instrument for goal attainment [39], *MYTS* Motivation for Youth’s Treatment Scale [40, 41], *TAQR* Therapeutic Alliance Quality Rating [40, 41], *YCIS v.2* Youth Counseling Impact Scale v.2 [40, 41], *TAQS* Therapeutic Alliance Quality Scale [40, 41], *OAS* German questionnaire to assess treatment adherence [42]

The online surveys are scheduled on a fixed basis and are conducted independently of the therapy sessions. As a consequence, they may be conducted even when no therapy session has taken place. However, in such instances, questionnaires that can be answered irrespective of a therapy session are used only (e.g., symptoms).

#### Content

The progress feedback system is based on questionnaires and, across all respondents, includes six domains: (1) symptoms, (2) goal attainment, (3) motivation, (4) treatment impact, (5) therapeutic alliance, and (6) compliance. Three of these (3, 4, 5) are from the Peabody Treatment Progress Battery [40], a progress feedback system, developed by Bickman and colleagues from the Vanderbilt University, whose questionnaires have in part been translated into German for the project [41]. It is based on a common factor approach and intends to address factors that are shared across different forms of psychotherapy (e.g., therapeutic alliance). The instruments have been psychometrically evaluated in a sample of American youths aged 11 to 18 years old receiving mental health care [40].

##### Symptoms

The Brief Problem Monitor for Ages 6–18 (BPM/6–18) [37, 38] is a 19-item measure with three subscales and a total scale (Internalizing, Externalizing, Attention Problems, Total). The forms for caregivers (BPM-P) and youths (BPM-Y) are short-forms of the Child Behavior Checklist for Ages 6–18 (CBCL/6–18) and the Youth Self-Report (YSR) [43]. They were developed to monitor a child’s functioning and their response to a treatment. The items are rated on a three-point Likert-type scale (0 = *not true* to 2 = *very true*) and scale scores are computed by the sum of the item scores. For the caregiver and the youth rating in a German clinical sample, reliability coefficients (Cronbach’s α) were between .72 and .83 [37]. German norm scores (*T* scores) are available based on age and gender [37].

##### Goal attainment

The German ZIEBO (Zielbeurteilungsbogen [Goal attainment form]) [39] monitors the attainment of the individual goals of the child (≥ 11 years) and the caregiver and has been adapted for the project. The goals should be developmentally appropriate, measurable, and achievable, and are defined during therapy. Each goal is rated standardized on a five-point Likert-type scale (− 1 = *worsened,* 0 = *unchanged,* + 1 = *slightly improved,* + 2 = *clearly improved*, + 3 = *considerably improved*). In addition to the goals themselves, each goal level from − 1 to + 3 should be defined individually as well. At the beginning of the therapy up to four goals may be determined and during the therapy up to four additional goals may be added. Each goal is evaluated individually and no scale score is computed.

##### Treatment motivation

The Motivation for Youth’s Treatment Scale (MYTS; German translation: Kinder- und Jugendlichen-Behandlungsmotivationsskala [KJBMS]) [40, 41] assesses intrinsic aspects of treatment motivation. The forms for youths (MYTS-Youth) and caregivers (MYTS-Adult Caregiver) both contain eight items that provide two subscales and a total scale (Problem Recognition, Treatment Readiness, Total). Items are rated on a five-point Likert-type scale (1 = *strongly disagree* to 5 = *strongly agree*) and the scale scores are the mean of the item scores. Reliability coefficients (Cronbach’s α) for the youth and the caregiver scales were between 0.82 and 0.88 [40]. Reference values are provided and may be used to evaluate the individual scale scores [40].

##### Treatment impact

The Youth Counseling Impact Scale v.2 (YCIS v.2; German translation: Therapiewirkungsskala [TWS]) intends to measure the positive effect of a single treatment session [40, 41] and is rated by the child only. Six items can be combined into two subscales and a total scale (Insight, Change, Total). The subscales aim to assess the immediate insights into the problems and solutions (Insight) as well as the cognitive, emotional, and behavioral changes after the last session (Change). Items are rated on a five-point Likert-type scale (1 = *not at all a problem* to 5 = *totally*) and scale scores are computed by the mean of the item scores. Reported reliability coefficients (Cronbach’s α) are between .82 and .90 and reference values are provided to facilitate the interpretation of the scores of an individual patient [40].

##### Therapeutic Alliance

The Therapeutic Alliance Quality Scale (TAQS; German translation: Therapiebeziehungsskala [TBS]) is a youth-rated measure to assess the relationship with the therapist and the agreement on the goals and tasks during therapy [40, 41]. Five items are combined into a single score by the mean of the item scores (Total). Additionally, the Therapeutic Alliance Quality Rating (TAQR; German translation: Therapiebeziehungsskala [TBS]) is applied to assess the quality of the therapeutic bond in a two-item form rated by the caregiver (TAQR-Adult Caregiver) and a four-item form rated by the therapist (TAQR-Clinician) [40, 41]. In both forms, the items are not aggregated and instead the items are interpreted in isolation. All items are rated on five-point Likert-type scale (TAQS: 1 = *not at all* to 5 = *totally*; TAQR: 1 = *very poor* to 5 = *excellent*). For the TAQS Total score the reported reliability coefficient (Cronbach’s α) is .85 and reference values are additionally provided [40].

##### Adherence

The German adherence scale OAS (OPTIE Adhärenzskala) is a clinician-rated measure to assess the willingness of the child and the caregiver to participate in the treatment and to follow the treatment recommendations [42]. It has been prepared for this project and is based on previous work [44]. In total, six items are rated on a four-point Likert-type scale (0 = *not at all* to 3 = *particularly*) and two scales are computed by the mean of the item scores (Child, Caregiver).

#### Progress report

**Fig. 2:**
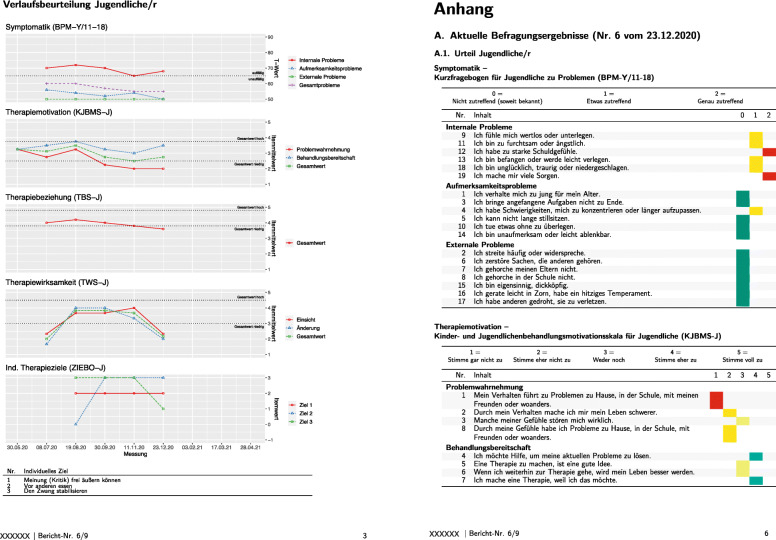
Excerpt from the German OPTIE progress report. Note. The left panel contains the progress charts of the child rating regarding five outcome domains (symptoms, motivation, therapeutic alliance, treatment impact, goal attainment). The right panel is part of the appendix and shows the individual item answers for the last assessment grouped by scales. Individual items scores are colored to increase readability

The progress report contains the results of the online surveys and is provided to the therapist and the supervisor after each online survey. It consists of four sections (title page, notes, progress charts, appendix; see Fig. 2). The title page contains basic information about the case (e.g., patient’s name and birthdate) and a note section mainly made up of organizational information is intended to facilitate the handling of the progress feedback system and increase the treatment integrity (e.g., date of next supervision). The progress charts display the trajectories over the period of the questionnaire data at the scale level. And, finally, the appendix contains the results of the questionnaire data at the item level, but only for the latest online survey.

#### Supervision

During the 12-month intervention period, therapists of the feedback condition receive five additional sessions of supervision lasting about 25 min each. These sessions are funded by the project. They are directed to support the application and the efficacy of the progress feedback system. The first session addresses the goal attainment scale ZIEBO and should take place 8 to 10 weeks after the start of therapy. The ZIEBO is part of the feedback system and therapists are requested to develop the personal goals with the patient and the caregivers at the beginning of the therapy. Under this supervision, the personal goals can be checked to ensure they are suitable for the therapy.

Supervision sessions two to five are intended to discuss the progress reports 3, 5, 7, and 9 from the online surveys 12, 24, 36, and 48 weeks after the start of the therapy, respectively, and to evaluate the treatment progress reports. During this supervision, it should be discussed if the treatment course is in line with the expectations of the therapist and the individual circumstances of a patient. In case of an unexpected or a negative trend, possible corrective measures should be discussed and the treatment planning should be adapted accordingly.

### Assessment

#### Schedule

For the group comparison (routine, feedback), data is collected at three assessments (pre, inter, post): at baseline before the beginning of the therapy as well as 6 and 12 months later (halfway through and at the end of the study-related intervention period).

#### Informants and blinding

For the assessments, five respondents are considered: (1) blinded clinician, (2) therapist, (3) caregiver, (4) child (≥ 11 years), and (5) teacher.

The blinded clinicians are study personnel and are responsible for the interviews with the caregivers. They are blinded for the treatment group (routine, feedback) and, whenever possible, also for the time of measurement (pre, inter, post), but this cannot be guaranteed. In general, interviews are conducted by telephone and are recorded whenever the caregivers give consent. There are two different means in the project of ensuring blinding for the treatment: one applies to interviewers who are involved in core processes of the study and who may have direct access to the project database, and the other applies to project staff with no direct access to the database and whose main responsibility is to conduct the interviews. To ensure blinding for the first group, the interview is organized by the project staff, and not by the person who is responsible for the interview. In this case, the interviewer is not aware of the identity of the telephone partner. For the second group, the interviewer may know the identity of the telephone partner (e.g., name), but will not know the treatment group. Either way, before the interview, the caregivers are requested not to reveal any sensitive information to the interviewer during the assessment (i.e., time of assessment, treatment group). In cases where the requirements for blinding of the treatment groups are not met, the audio record will be edited (when necessary) and will be rated again by a different blinded clinician. To ensure blinding for the time of the assessment, it is intended that a caregiver is interviewed by the same interviewer only once. However, for organizational reasons, the pre-assessment is difficult to blind and, in some instances, multiple ratings by the same interviewer for one family may occur.

The therapist is the practitioner responsible for the therapy. Caregivers refers to biological parents or guardians who support the child during therapy. The children, of 11 years and older, are the patients in the therapy. The study also includes younger children from the age of 6 years old. However, reports from those under the age of 11 are not considered for reasons of feasibility, reliability, and validity. The teacher may be the class teacher of the child or any other teacher with a more detailed knowledge of the behavior of the child. The teacher assessment will be conducted only if the caregiver and the child give their consent.

### Measures

As diagnostic measures, an interview and several questionnaires are used. The interview is rated by the blinded clinician. The questionnaires may be rated by different respondents depending on the particular instruments (i.e., blinded clinician, therapist, caregiver, child [≥ 11 years], teacher). All measures may be assessed up to three times (see Table 2). The primary outcome parameter is the total score of the clinical interview. Secondary outcome parameters may include all instruments measured at the post-assessment.

**Table 2:** Measures of the pre-, inter-, and post-assessment at baseline and at 6 and 12 months after the beginning of the therapy

Measure	Domain	Assessment		
		Pre	Inter	Post
Blinded clinician				
SFSS-I	Symptoms – child	X	X	X
GBPF	Functioning – child	X	X	X
Therapist				
BADO	Basic documentation	X		X
GBPF	Functioning – child	X	X	X
FBB	Treatment satisfaction			X
Caregiver				
TOES	Outcome expectations	X		
CBCL/6–18 or BPM/6–18^a^	Symptoms – child	X	X	X
FBB-SCREEN	Symptoms and impairment – child	X	X	X
KIDSCREEN	Quality of life – child	X	X	X
FBB	Treatment satisfaction			X
Child (≥ 11 years)				
TOES	Outcome expectations	X		
YSR or BPM/6–18^a^	Symptoms – child	X	X	X
SBB-SCREEN	Symptoms and impairment – child	X	X	X
KIDSCREEN	Quality of life – child	X	X	X
FBB	Treatment satisfaction			X
Teacher				
TRF	Symptoms – child	X		X

*Note*. *SFFS-I* Symptoms and Functioning Severity Scale – Interview version (SFSS-I) [45], *GBPF* German rating scale for the assessment of the psychosocial functioning [46], *FBB* German questionnaire for treatment satisfaction [47], *BADO* German basic assessment system for psychotherapy [48], *TOES* Treatment Outcome Expectations Scale [40], *CBCL/6–18* Child Behavior Checklist for Ages 6–18 [43, 49], *BPM/6–18* Brief Problem Monitor for Ages 6–18 [37, 38], *FBB-SCREEN* German screening questionnaire for mental disorders and impairment [50]; *KIDSCREEN* Questionnaire for health-related quality of life [51], *YSR* Youth Self-Report, *SBB-SCREEN* German screening questionnaire for mental disorders and impairment [50], *TRF* Teacher Report Form [43, 49]

^a^BPM forms are short-forms of the CBCL/6–18 and YSR. The long-forms are used at pre- and post-assessment, the short-forms at the inter-assessment

#### Basic documentation

##### BADO

The German BADO (Basisdokumentation [Basic documentation]) [48] is a standardized measure for the assessment of essential data in psychotherapy and is rated by the therapist. The BADO admission form includes relevant information for the case preparation at the beginning of the therapy (e.g., social demographics, initial diagnosis, previous treatment) and the discharge form summarizes the treatment episode at the end of the treatment (e.g., final diagnosis, interventions during treatment) and includes recommendations for further treatment.

#### Treatment expectations

##### Treatment Outcome Expectations Scale (TOES)

The TOES (German translation: Therapieerwartungsskala [TES]) measures the anticipated treatment outcome and has forms for youths (TOES-Youth) and caregivers (TOES-Adult Caregiver) [40, 41]. Both forms contain eight items that are rated on a three-point Likert-type scale (1 = *I do not expect this* to 3 = *I do expect this*) and can be combined into a single score by the mean of the item scores (Total). Reported reliability coefficients (Cronbach’s α) are .91 for youths and .85 for adults [40]. The TOES may be used as a predictor for moderator analyses.

#### Symptoms – child

##### Symptoms and Functioning Severity Scale – Interview version (SFSS-I)

The SFSS-I is a newly developed 24-item semi-structured clinical interview in German [45]. All items stem from the Symptoms and Functioning Severity Scale (SFSS), which is part of the progress feedback system Peabody Treatment Progress Battery [40]. The SFSS is a global measure for symptom severity. It has been developed under consideration of the most common mental disorders in children and adolescents (i.e., attention-deficit/hyperactivity disorder, conduct disorder, oppositional defiant disorder, depression, and anxiety). In its original form, the SFSS is a questionnaire with forms for youths, caregivers, and clinicians that assess the progress feedback during treatment. The 24-item measure provides three scales (Internalizing, Externalizing, Total). All scales have shown good to excellent reliability (Cronbach’s α ≥ .88) [40].

There were four main reasons to develop the SFSS-I interview based on the items of the SFSS. First, the SFSS is a relatively brief measure that can be usefully applied under conditions of routine care. Second, the SFSS addresses broad domains of psychopathology (internalizing, externalizing). This is in line with the aim of the study and its sample: to analyze the efficacy of a progress feedback system in a general clinical sample of children and adolescents who exhibit either internalizing or externalizing problems but who have not been screened for a particular disorder. Third, the SFSS allows for a dimensional diagnostic approach (in contrast to a categorical approach) [52] and can be analyzed accordingly. Fourth, to the best of our knowledge, no other instrument satisfies the aforementioned conditions and can be applied to children aged 6 to 17 years old.

A manual was created during the development of the interview. It contains (1) the SFSS items themselves, (2) a newly developed detailed description of each item (i.e., meaning), and (3) a newly developed individual description of each item level. The additional item information is provided for the interviewer to clarify the content of the items and for exploration during the interview. The additional item level information is meant to increase the reliability of the item ratings. It was adapted from the German ILF (Interview-Leitfäden [Interview guidelines]) [53], a collection of semi-structured interviews for the assessment of disorders in children and adolescents. The rating scale of the SFFS-I was also adopted from the ILF, meaning that we used a four-point Likert-type scale (0 = *not presented*, 1 = *sometimes*, 2 = *often*, 3 = *very often*) instead of the original five-point scale of the SFSS (1 = *never*, 2 = *hardly ever*, 3 = *sometimes*, 4 = *often*, 5 = *very often*). This change was made given that the ILF interviews have shown good psychometric properties [54]. It is intended to adopt the scales of the SFSS for the SFSS-I (Total, Internalizing, Externalizing). The SFSS-I will be psychometrically evaluated before the scale scores are used [55]. The interview is rated by the blinded clinician and is conducted with the caregiver. It is the primary outcome parameter of the study.

##### ASEBA school-age Forms & Profiles (CBCL/6–18, YSR, TRF) and Brief Problem Monitor for Ages 6–18 (BPM/6–18)

The school-age forms measure the strengths and problems of children and youths [43, 49]. Versions for caregivers (Child Behavior Checklist for Ages 6–18 [CBCL/6–18]), youths (Youth Self-Report Form [YSR]), and teachers (Teacher’s Report Form [TRF]) are available and the study will mainly focus on three scales (Internalizing, Externalizing, Total Problems). Items are rated on a three-point Likert-type scale (0 = *not true* to 2 = *very true or often true*) and scale scores are computed by the sum of the item scores. For the scales under consideration in a German clinical sample, reliability coefficients (Cronbach’s α) were between .80 and .93 [49]. All three forms (CBCL/6–18, YSR, and TRF) will be used at pre- and post-assessment (see Table 1). At inter-assessment for parent and youth ratings, the BPM/6–18 short-forms for parents (BPM-P) and youths (BPM-Y) will be used instead (for instrument details see Section OPTIE Progress Feedback System) [37, 38].

##### FBB-SCREEN and SBB-SCREEN

The caregiver-rated FBB-SCREEN (Fremdbeurteilungsbogen zum Screening psychischer Störungen [Proxy-rating form for the screening of mental disorders]) and youth-rated SBB-SCREEN (Selbstbeurteilungsbogen zum Screening psychischer Störungen [Self-rating form for the screening of mental disorders]) are part of a German assessment system for mental disorders in children and adolescents [50] and are used to identify high-risk children and adolescents regarding mental health problems. Both forms contain 51 symptom items that are rated on a four-point Likert-type scale (0 = *not at all* to 3 = *very much*). Several symptom scales can be computed by the mean of the item scores (e.g., Conduct Disorder, Anxiety, Depression, Total). For most of the scales the reliability coefficients (Cronbach’s α) of both forms have been between .67 and .92 [50].

#### Functioning and impairment – child

##### GBPF

The German GBPF (Globale Beurteilung des psychosozialen Funktionsniveaus [Global assessment of psychosocial functioning]) is part of a German multi-axial classification scheme of mental disorders in children and adolescents based on ICD-10 [46]. The GBPF is a one-item clinical rating scale that measures functioning (e.g., psychological, social, school/occupational) due to a psychiatric disorder. Its item levels range from 0 (*excellent or good social adaptation in all areas*) to 8 (*needs constant care [24/7]*).

##### FBB-SCREEN and SBB-SCREEN

Both the caregiver-rated FBB-SCREEN and the youth-rated SBB-SCREEN contain five items to measure functional impairment and psychological strain [50]. The items are scored on a four-point Likert-type scale (0 = *not at all* to 3 = *very much*) and can be combined into a single scale by the mean of the item scores (Impairment).

#### Quality of life

##### KIDSCREEN-10 Index

The KIDSCREEN-10 Index measures health-related quality of life in children and adolescents [51] and provides forms for caregivers and youths. The items cover physical, psychological, and social aspects and are rated on a five-point Likert-type scale (1 = *not at all/never* to 5 = *extremely/always*). Both forms contain 10 items that each provide a single scale (Total). For the self-report form, a reliability coefficient (Cronbach’s α) of .82 has been reported [51].

#### Treatment satisfaction

##### FBB

The German FBB (Fragebogen zur Beurteilung der Behandlung [Questionnaire for the treatment assessment]) measures treatment satisfaction with a focus on the treatment process and the outcome quality of the therapy [47]. Forms for youths (FBB-P), caregivers (FBB-E), and therapists (FBB-T) contain 20, 21, and 26 items, respectively, and are rated on a five-point Likert-type scale (0 = *not at all/never* to 4 = *exactly/always*). A total scale as well as several subscales are provided. Scale scores are computed by the mean of the item scores. Depending on the form and the scale reported, reliability coefficients (Cronbach’s α) were between .71 and .94 [47].

## Methods and statistics

### Planned sample size and power calculations

For the sample size determination an effect size in the small to medium range was hypothesized. The calculation was done with Stata/SE (StataCorp LLC, College Station, TX, USA) under the presumption that the treatment groups (routine, feedback) would be compared using a two-sample *t*-test. The α-error was specified to be .05, the power to be .80, and the effect size (Cohen’s *d*) equal to .30. With these assumptions, the required sample size was 351 families. To compensate for attrition during the study (e.g., withdrawal of consent, treatment drop-out), the inclusion of 20% more patients than the required sample size was planned, resulting in a total sample size of 439 families.

### Intention-to-treat and per-protocol sample

For the analyses, two samples are distinguished (intention-to-treat, per-protocol). The intention-to-treat sample refers to all patients randomized (both regular and forced) to the study. Families remain part of the intention-to-treat sample irrespective of any irregularities or violations of the protocol after the randomization process (e.g., unregular treatment participation, early treatment termination). In contrast, the per-protocol sample consists of families without any major violations of the study protocol.

### Data management

The project data will be stored in secure databases that are either part of the routine care or have been specifically set up for the project. Access to these databases is only granted to the project staff or members of the University Hospital Cologne who are authorized and involved in the care of the patient. Data control routines will be established to ensure data quality (e.g., value range checks). For this trial there are no regulations or reasons to necessitate the involvement of a data monitoring committee or an external auditing process. Project staff meetings will be held and instructions will be given on a regular basis to monitor the conduct of the trial and to ensure the quality of the data.

### Statistical analysis

The main purpose of the study is to evaluate the effectiveness of the progress feedback system and the primary analysis will concern the comparison of the feedback group and the routine group. Data will be analyzed by linear mixed-effects models [56]. All three assessments will be included (pre, inter, post) and the outcome at the pre-assessment will be used to adjust for baseline differences in the treatment groups [57]. Both the intention-to-treat as well as the per-protocol samples will be analyzed. However, the intention-to-treat analysis is considered the method of choice to preserve the integrity of the randomization process [58]. The secondary analyses will concern the participation behavior in the feedback group, the treatment drop-out, moderator and mediator analyses, and more.

Missing data will be analyzed, and recommended methods for adjustments will be applied (e.g., multiple imputation) [59]. Additionally, sensitivity analysis will be conducted to test the robustness of the assumptions when handling missing data.

## Discussion

The main goal of the OPTIE study is to develop and evaluate a progress feedback system for children and adolescents aged 6 to 17 years old with internalizing and externalizing symptoms under the conditions of routine care. The feedback system contains three elements (monitoring, report, supervision). Over a 12-month intervention period, caregivers, youths, and therapists are asked to answer brief online surveys every 6 weeks that may cover up to six different domains (i.e., symptoms, goal attainment scaling, motivation, treatment impact, therapeutic alliance, and compliance). The resulting report contains the results of the online surveys and is given to the therapist and the supervisor. The five sessions of supervision are intended for the discussion of the treatment course and to adapt the treatment planning when necessary.

The progress feedback system is tested by a randomized parallel-group design with two treatment arms – routine group and feedback group – and with a planned sample size of 439 families. A multi-informant approach is followed and includes five perspectives. The primary outcome is the symptom severity of the child rated by the blinded clinician and the primary analysis will test if the feedback group has a treatment advantage compared to the routine group.

The study has the strengths of a large intended sample size and may have the potential to uncover unbiased treatment effects in the small to medium range. The trial may further be valuable because children younger than 10 years old are included and a blinded clinician rating is considered – features that are rarely found in previous studies [30]. The multi-informant approach may also be important, as each respondent may contribute to the understanding of the child’s symptoms [60]. In addition to the effects in the total group, subgroups of patients are of importance as well. Moderator analyses have the potential to not only help identify patients with a particularly positive treatment response but also help identify those who may show no improvement.

The implementation of the study may be challenged by the conditions of routine care. One area of concern is the adherence of the participants to the progress feedback system as there is a known risk for low response rates [61]. Therefore, it is considered vital to establish a monitoring system and to identify possible obstacles for participation at an early stage, as well as to initiate compensatory measures when needed. In addition to the challenges of the routine care setting there may also be some advantages. Only a minimum amount of inclusion criteria is defined and a diverse sample is collected. In addition, the therapies are conducted under real-world conditions. The findings of the study may therefore be more easily generalized to other settings [62].

Progress feedback systems are rarely investigated among children and adolescents and the results of this study will contribute to the evidence base of the approach for this particular age group. Findings from moderator analyses may help to develop guidelines concerning under which circumstances progress feedback may be particularly useful. If proven effective, the progress feedback system will support therapists in their daily work and help to adapt treatment planning to the changing needs of the patient as well as improve communication with the patient.

## Data Availability

The pseudonymized final dataset and the full study protocol including related materials may be made available upon request by the principal investigator.

## Abbreviations

AKiP: School for Child and Adolescent Psychotherapy at the University Hospital Cologne
BADO: German clinician-rated questionnaire for the basic documentation in psychotherapy
BPM/6–18: Brief Problem Monitor for Ages 6-18 (BPM/6–18) with forms for youths (BPM-Y) and parents (BPM-P)
BPtK: Federal Chamber of Psychotherapists in Germany
CASCAP-D: German clinician-rated assessment scale for child and adolescent psychopathology
CBCL/6–18: Child Behavior Checklist for Ages 6–18
DSG NRW: North Rhine-Westphalia Data Protection Act
FBB: German questionnaire for treatment satisfaction with forms for therapists (FBB-T), caregivers (FBB-E), and youths (FBB-P)
FBB-SCREEN: German caregiver-rated questionnaire for the screening of mental disorders and impairment in children and adolescents
G-BA: Federal Joint Committee
GBPF: German clinical rating scale for the assessment of the psychosocial functioning of the child
KIDSCREEN: Questionnaire for the assessment of health-related quality of life in children and adolescents with forms for parents and children
MYTS: Motivation for Youth’s Treatment with forms for youths (MYTS-Youth) and caregivers (MYTS-Adult Caregiver)
OAS: German clinician-rated questionnaire for the assessment of the adherence of the child and the caregiver
OPTIE: Acronym of this study
REDCap: Research Electronic Data Capture
SBB-SCREEN: German youth-rated questionnaire for the screening of mental disorders and impairment
SFSS: Symptoms and Functioning Severity Scale
SFSS-I: Symptoms and Functioning Severity Scale – Interview version
TAQR: Therapeutic Alliance Quality Rating
TAQS: Therapeutic Alliance Quality Scale
TOES: Treatment Outcome Expectations Scale; forms for youths (TOES-Youth) and caregivers (TOES-Adult Caregiver) are available
TRF: Teacher Report Form
YCIS v.2: Youth Counseling Impact Scale v.2
YSR: Youth Self-Report Form
ZIEBO: German instrument for goal attainment scaling
